# Delayed Presentation of Congenital Diaphragmatic Hernia in the Emergency Department: Case Report

**DOI:** 10.5811/cpcem.41565

**Published:** 2025-06-19

**Authors:** Morsi Rayyan, Ryan Reece

**Affiliations:** * University of Michigan, Department of Emergency Medicine, Ann Arbor, Michigan; † University of Michigan, Hurley Medical Center, Department of Emergency Medicine, Flint, Michigan

**Keywords:** delayed presentation, congenital diaphragmatic hernia, respiratory distress, case report

## Abstract

**Introduction:**

Congenital diaphragmatic hernia (CDH) is an embryological defect of the diaphragm that typically presents in the neonatal period with respiratory distress. However, delayed presentations do occur rarely and can pose diagnostic and therapeutic challenges.

**Case Report:**

We describe the case of a 9-month-old male who presented to the emergency department (ED) with respiratory distress and was subsequently diagnosed with congenital diaphragmatic hernia.

**Conclusion:**

This case underscores the importance of considering CDH in the differential diagnosis of pediatric patients presenting to the ED with unexplained respiratory or gastrointestinal symptoms, even beyond the neonatal period.

## INTRODUCTION

Congenital diaphragmatic hernia (CDH) occurs due to a developmental defect in the diaphragm, allowing abdominal contents to herniate into the thoracic cavity. This condition predominantly presents in neonates with severe respiratory distress immediately after birth. Delayed presentation beyond the neonatal period is rare and often misdiagnosed due to the non-specific nature of symptoms. Early recognition and appropriate management are crucial to improving outcomes. In this case report we aimed to increase awareness among emergency physicians about the potential for delayed CDH presentation and highlight the importance of thorough clinical evaluation and timely intervention in pediatric patients with persistent respiratory distress.

## CASE REPORT

A nine-month-old male presented to the emergency department (ED) with respiratory distress and cyanosis. The patient’s parent reported that the patient began crying and his lips appeared blue. Emergency medical services who responded noted that the patient had wheezing bilaterally with retractions and an oxygen saturation of 86%. He was given nebulized ipratropium-albuterol and blow-by oxygen, and transported to the ED. Of note, he had been hospitalized at three weeks of age for acute hypoxic respiratory failure in the setting of respiratory syncytial virus and human rhinovirus-enterovirus infection requiring pediatric intensive care and high-flow oxygen. He had a chest radiograph (CXR) at the time, which showed bilateral hazy opacities suggesting viral process or small airway disease. His parents reported intermittent episodes of coughing and wheezing since then that had worsened over the previous two days. His immunizations were up to date, and he had no history of other infectious or gastrointestinal (GI) symptoms, or trauma. He had been born at term following an uncomplicated pregnancy. Of note, the patient was at the third percentile for weight.

On arrival to the ED, the patient was found to have a blood pressure of 77/54 millimeters of mercury (mm Hg), heart rate 183 beats per minute, respiratory rate 28 breaths per minute, oxygen saturation 99% on blow-by oxygen, and oral temperature of 37.3 ºCelsius. Physical exam revealed increased work of breathing with intercostal retractions and diminished air entry bilaterally with faint expiratory wheezing. Initial labs were significant for a venous blood gas with pH 7.33 (reference range: 7.30–7.40), partial pressure of carbon dioxide 49 mm Hg (33–46 mm Hg) and a metabolic panel significant for bicarbonate of 22 milliequivalents per liter (mEq/L) (23–29 mEq/L). A CXR showed bubbly lucencies in the left hemithorax with rightward mediastinal shift and adjacent compressive atelectasis of the right lung, suspicious for large diaphragmatic hernia with bowel throughout the left hemithorax (Image).

The patient was initially treated with additional nebulized ipratropium-albuterol and four milligrams dexamethasone for wheezing while diagnostics were being performed, with concern for reactive airway disease. His work of breathing did not improve considerably. After the CXR was available, a nasogastric tube was considered for gastric decompression, but appropriately sized pediatric tubes were unavailable. The patient’s case was discussed with a pediatric tertiary-care center, and he was transferred. There, he underwent left open CDH repair.

*CPC-EM Capsule*

What do we already know about this clinical entity?

*Congenital diaphragmatic hernia (CDH) usually presents neonatally with respiratory distress; delayed presentations are rare but can occur*.

What makes this presentation of disease reportable?

*It highlights a rare delayed CDH presentation diagnosed in the emergency department, missed on prior imaging, emphasizing diagnostic vigilance*.

What is the major learning point?

*CDH should be considered in pediatric patients with recurrent respiratory symptoms unresponsive to standard treatments*.

How might this improve emergency medicine practice?

*Raises awareness of delayed CDH presentation and reviews diagnostic and management strategies for emergency physicians*.

The patient had a postoperative course briefly complicated by an expected pneumothorax and desaturations that improved with blow-by oxygen. He was discharged on postoperative day three. At his six-week follow-up, he was noted to be doing well with improved respiratory symptoms and oral intake. His weight had improved to the 38^th^ percentile.

## DISCUSSION

Congenital diaphragmatic hernia is thought to be caused by multiple factors including genetics, environmental exposures, and nutritional deficiencies, all resulting in incomplete diaphragmatic development. The diaphragmatic defect allows intra-abdominal organs to migrate into the thoracic cavity, which can impede normal lung development and function. Congenital diaphragmatic hernia has an incidence ranging from 0.8–5/10,000 live births and is most commonly diagnosed prenatally or immediately postnatally.^1^ Most CDH cases present in neonates with severe respiratory distress necessitating immediate intervention. Delayed presentations, such as in the case we describe, are rare and account for an estimated 10–13% of all CDH cases. These cases often present with non-specific symptoms including recurrent respiratory infections, GI symptoms, and failure to thrive.^2^

In older infants and children, the delayed presentation of CDH can mimic common pediatric conditions such as asthma, pneumonia, or gastroesophageal reflux, leading to misdiagnosis and delayed treatment. Key clinical features that should raise suspicion for CDH in the differential diagnosis include the following: persistent or recurrent respiratory symptoms unresponsive to standard medical treatments; unilateral decreased breath sounds or abnormal chest auscultation findings; GI symptoms such as vomiting, feeding difficulties, or failure to thrive; and abnormal CXR findings including air-fluid levels or mediastinal shift.^3^–^5^

Diagnosis of CDH in delayed presentations often relies on imaging studies. A CXR is typically the initial imaging modality and may reveal bowel loops in the thoracic cavity, an elevated hemidiaphragm, or mediastinal shift. Chest radiograph is warranted even if the patient has had a prior radiograph that did not show CDH, as in the presented patient. In cases of diagnostic uncertainty, a nasogastric tube can be placed with subsequent contrast administration and follow-up CXR to aid in diagnosis. Additionally, abdominal radiographs, small bowel follow-through, and abdominal and thoracic ultrasound may lead to CDH diagnosis. Further imaging with contrast-enhanced computed tomography, the most sensitive modality, can provide detailed anatomy, confirm the diagnosis, and assist in preoperative planning by delineating the size and contents of the hernia.^6^

Definitive management of CDH presenting beyond the neonatal period involves surgical correction of the hernia and repair of the diaphragmatic defect. Preoperative stabilization is often necessary in the ED, including respiratory support and nasogastric decompression, especially in cases with significant respiratory compromise. Emergency physicians should be conscious of the potential for positive pressure ventilation, such as that provided by a bag valve mask, to cause gastric distention and worsen respiratory instability. They should perform endotracheal intubation in severe respiratory compromise or if prolonged resuscitation is required, similar to the suggested management in neonatal CDH.^1^ Additionally, the presented case highlights the importance of stocking appropriately sized pediatric equipment as nasogastric decompression could not be performed in a timely manner and could have led to decompensation prior to definitive management. In the event of failed nasogastric decompression leading to refractory respiratory failure, cases have been described using bedside percutaneous gastric puncture, emergent thoracotomy, and initiation of extracorporeal membrane oxygenation, although we recommend considering these approaches only in consultation with a pediatric surgeon.^7^–^9^

The prognosis for patients with delayed presentation of CDH is favorable if timely diagnosis and surgical intervention are achieved. However, the severity of pulmonary hypoplasia and any associated congenital anomalies can influence outcomes. Long-term follow-up is recommended to monitor for respiratory function, growth, and development as well as to identify and address any late complications.^2^,^3^,^10^

## CONCLUSION

Congenital diaphragmatic hernia is a rare but important differential diagnosis for pediatric patients presenting with unexplained respiratory distress, particularly when symptoms are refractory to standard treatments. This case report highlights the necessity for emergency physicians to maintain a high index of suspicion for CDH in older infants and children with persistent respiratory and gastrointestinal symptoms. Early recognition and appropriate referral for surgical management are critical to improving outcomes in these patients.

## Figures and Tables

**Image:**
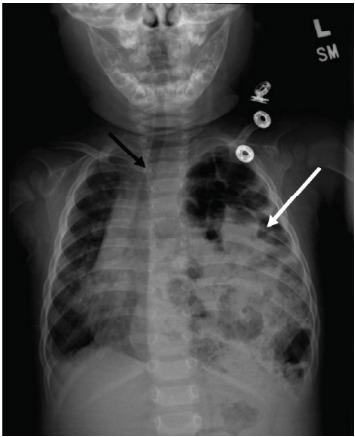
Chest radiograph with white arrow indicating an area of herniated bowel throughout the left hemithorax and black arrow indicating rightward tracheal deviation and mediastinal shift.
